# Note on the genus *Serendib* Deeleman-Reinhold, 2001, with the description of a new species (Araneae, Corinnidae, Castianeirinae)

**DOI:** 10.3897/BDJ.11.e99980

**Published:** 2023-02-15

**Authors:** Lu Zhang, Feng Zhang

**Affiliations:** 1 The Key Laboratory of Zoological Systematics and Application, Institute of Life Science and Green Development, College of Life Sciences, Hebei University, Baoding, China The Key Laboratory of Zoological Systematics and Application, Institute of Life Science and Green Development, College of Life Sciences, Hebei University Baoding China

**Keywords:** Dionycha, morphology, spiders, taxonomy

## Abstract

**Background:**

Prior to this study, the genus *Serendib* Deeleman-Reinhold, 2001 has been discovered in Indonesia, Thailand and Laos and comprises three species: *S.muadai* Jäger, Nophaseud & Praxaysombath, 2012, *S.suthepica* Deeleman-Reinhold, 2001 and *S.volans* Deeleman-Reinhold, 2001.

**New information:**

The genus *Serendib* Deeleman-Reinhold, 2001 is reviewed. A new species, *S.hispida* **sp. n.** (male and female), representing the first record of genus *Serendib* from Malaysia, is described. Descriptions and illustrations of the females of *S.volans* (Malaysia) and *S.suthepica* (China) are also provided. The latter represents the first record of the genus in China.

## Introduction

Deeleman-Reinhold (2001) erected the genus *Serendib*, based on the type species *Serendibvolans* Deeleman-Reinhold, 2001 and placed it in Castianeirinae, Corinnidae. Meanwhile, another species *S.suthepica* Deeleman-Reinhold, 2001 was described somatically for both sexes, but without detailed descriptions of genital characteristics (Deeleman-Reinhold 2001). Subsequently, the third species, *S.muadai* Jäger, Nophaseud & Praxaysombath, 2012 was described and illustrated (Jäger et al. 2012).

While examining the corinnids specimens collected from the Oriental Region during the past decade, we found one species that is consistent with the generic characteristics of *Serendib*, namely *S.hispida* **sp. n.**, representing the first record of *Serendib* from Malaysia. The species, *Serendibvolans* and *S.suthepica* are redescribed, with the latter recorded from China for the first time, representing the northernmost record for *Serendib*. Descriptions and illustrations are provided for the females of two known species and both sexes of one new species.

## Materials and methods

All measurements are given in millimetres (mm). Leg measurements are shown as total length (femur, patella, tibia, metatarsus, tarsus). Epigynes were removed and cleared in a pancreatin solution (Álvarez-Padilla and Hormiga 2007). All specimens are preserved in 95% alcohol and were examined, illustrated and measured with a Leica M205A stereomicroscope. Somatic photographs were captured using a Leica M205A stereomicroscope, equipped with a DFC550 CCD camera and morphology photographs were taken using an Olympus BX51 microscope equipped with a Kuy Nice CCD with a Canon 60 mm micro-lens and were imported into Helicon Focus 7 for stacking. Drawings were used with Inkscape version 1.0.2.0. Final figures were retouched with Adobe Photoshop CC © 2022. The distribution map was made using ArcGIS Desktop version 10.6. The specimens used in this study are deposited in the Museum of Hebei University, Baoding, China (MHBU).

The abbreviations used in the text are as follows: Eyes: ALE = anterior lateral eye; AME = anterior median eye; MOA = median ocular area; PLE = posterior lateral eye; PME = posterior median eye; RTA = retrolateral tibial apophysis.

## Taxon treatments

### Serendib

Deeleman-Reinhold, 2001

5602E82E-010B-5C8D-8D42-9A6F2FEA19CC

Serendib Serendib volans Deeleman-Reinhold, 2001

#### Description

Small spiders, 4–6 mm in length. Carapace wedge-shaped or elongated, smooth or with plumose hairs; several long setae on clypeus and eye region; broadest of carapace at coxae Ⅱ–Ⅲ (Fig. 7a); anterior eye row slightly recurved in dorsal view and posterior eye row very wide and strongly recurved (Fig. 1a); fovea short, longitudinal, indistinct. Chelicerae same colour as carapace, both promargin and retromargin with two teeth. Sternum about as broad as long, slightly narrowed posteriorly. Leg formula 4123; femora Ⅰ–Ⅱ with one row of long bristles ventrally (usually four or eight), femora Ⅲ–Ⅳ with one or two long bristles ventrally (Fig. 3). Abdomen round or oval in females, elongate oval in males; with short or long grooved collar (Fig. 1b–d); anterior with one or two pairs of strongly erected spines (Fig. 2); dorsal scutum strongly sclerotized (Fig. 7a).

Palpal tibia longer than wide, with retrolateral hump and small RTA. Subtegulum visible in retrolateral view. Sperm duct coiled and formed loops, anterior with extra transverse U-shaped loop in *S.hispida* **sp. n.** and *S.suthepica*. Embolus with distinctly narrowed tip (Fig. 8a–d and fig. 517 in Deeleman-Reinhold (2001)).

**Figure 4.**
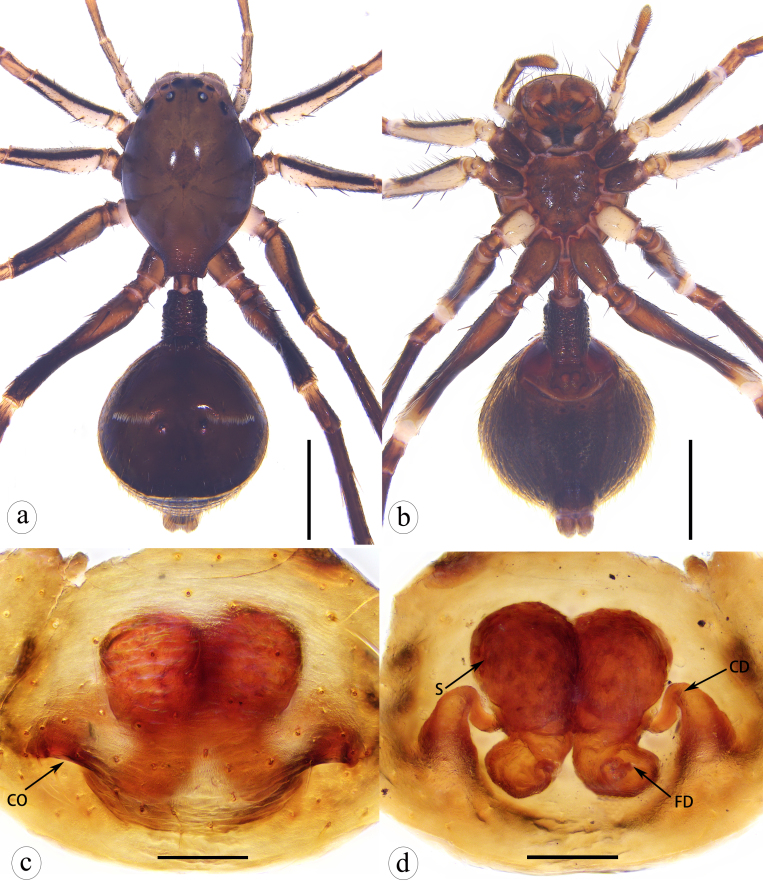
*Serendibvolans* Deeleman-Reinhold, 2001: **a** female habitus, dorsal view; **b** same, ventral view; **c** epigyne, ventral view; **d** vulva, dorsal view. Abbreviations: CO—copulatory opening; CD—copulatory duct; FD—fertilization duct; S—spermatheca. Scale bars: 1 mm (a–b); 0.1 mm (c–d).

Epigyne simple and strongly sclerotized. Copulatory openings posteriorly situated, with simple sclerotized margins. Copulatory ducts gradually extended into anterior spermathecae. Posterior spermathecae separated or contiguous (Fig. 4c–d; Fig. 6c–d; Fig. 8e–f).

#### Diagnosis

All species of *Serendib* resemble those of *Sphecotypus* O. Pickard-Cambridge, 1895 and *Aetius* O. Pickard-Cambridge, 1897 in ant-mimicking, small body size and having wide, strongly recurved posterior eye row (Fig. 1a), but can be distinguished by the following characters: 1) abdomen without transverse constriction (Fig. 4a–b; Fig. 6a–b; Fig. 7a, c); 2) anterior abdomen with one or two pairs of strongly erected spines (Fig. 2); 3) femora Ⅰ–Ⅱ with one row of long bristles ventrally (usually four or eight), femora Ⅲ–Ⅳ with one or two long bristles ventrally (Fig. 3); 4) copulatory duct long, curved or spiral (Fig. 4d; Fig. 6d; Fig. 8f).

#### Distribution

China, Indonesia, Laos, Malaysia, Thailand (Fig. 9).

### Serendib volans

Deeleman-Reinhold, 2001

50AB85C1-BCCB-5E31-A992-F2A22E4B9E36

#### Materials

- **Type status:** Other material. **Occurrence:** recordedBy: Chi Ji; individualCount: 1; sex: female; lifeStage: adult; occurrenceID: 97B26255-0FE4-505D-8BE4-85D25C017548; **Taxon:** scientificName: *Serendibvolans* Deeleman-Reinhold, 2001; **Location:** country: Malaysia; stateProvince: Negeri Sabah; locality: Forest Girl Camp; **Event:** year: 2017; month: 5; day: 2; **Record Level:** institutionID: the Museum of Hebei University; institutionCode: MBHU

#### Description

Female (Fig. 4a–b). Total length 5.32; carapace 2.07 long, 1.44 wide; abdomen 3.25 long, 1.76 wide. Eye sizes and interdistances: AME 0.13, ALE 0.10, PME 0.11, PLE 0.09; AME–AME 0.22, AME–ALE 0.14, ALE–ALE 0.47, PME–PME 0.29, PME–PLE 0.21, PLE–PLE 0.65, ALE–PLE 0.18. MOA 0.22 long, anterior width 0.31, posterior width 0.37. Clypeal height 0.13. Labium 0.12 long, 0.31 wide. Sternum 0.93 long, 0.81 wide. Measurements of legs: I 5.18 (1.46, 0.46, 1.22, 1.11, 0.93), II 5.01 (1.44, 0.47, 1.17, 1.05, 0.88), III 4.84 (1.43, 0.45, 1.12, 1.11, 0.73), IV 6.32 (1.83, 0.49, 1.53, 1.55, 0.92).

Carapace brown, with smooth surface (Fig. 4a). Legs slender, brown; femora I–II black, with two white and longitudinal stripes (Fig. 4a–b); femora I–II with long bristles ventrally (Fig. 3c). Abdomen dark brown and round, with a pair of sigilla; with long and transverse groove collar (Fig. 4a; Fig. 1d); anteriorly with one pair of strongly erected spines (Fig. 2b, d). Dorsal scutum large, round, nearly covered abdomen, with a pair of white stripes (Fig. 4a).

Epigyne as in Fig. 4c–d. Copulatory openings distinct, with arc-shaped sclerotized margins. Copulatory ducts basal wide, gradually narrowed and extended into anterior spermathecae.

#### Diagnosis

The female of *S.volans* resembles that of *S.muadai* in having similar colouration and habitus, but can be distinguished by the following characteristics: 1) abdomen with a pair of sigilla (vs. absent in *S.muadai*) (cf. Fig. 4a with fig. 55 in Jäger et al. (2012)); 2) posterior spermathecae oval, distinctly separated (vs. posterior spermathecae round, closed to each other in *S.muadai*) (cf. Fig. 4d with fig. 45 in Jäger et al. (2012)).

#### Distribution

Thailand, Malaysia (Borneo) (Fig. 9).

### Serendib suthepica

Deeleman-Reinhold, 2001

39E593FF-1125-58CE-99F1-22261BA7A257

#### Materials

- **Type status:** Other material. **Occurrence:** recordedBy: Kun Yu; individualCount: 3; sex: female; lifeStage: adult; occurrenceID: A4BFC04E-8B8F-5140-962E-E66E4586DDC2; **Taxon:** scientificName: *Serendibsuthepica* Deeleman-Reinhold, 2001; **Location:** country: China; stateProvince: Yunnan Province; county: Jinghong; locality: Wild Elephant Valley; verbatimElevation: 800m; verbatimLatitude: 22°10′24.12″N; verbatimLongitude: 100°51′33.75″E; **Event:** year: 2021; month: 8; day: 4; **Record Level:** institutionID: the Museum of Hebei University; institutionCode: MHBU
- **Type status:** Other material. **Occurrence:** recordedBy: Lu Zhang; individualCount: 1; sex: female; lifeStage: adult; occurrenceID: 5395DD71-5DB3-51C6-ACEA-D2193C1D1651; **Taxon:** scientificName: *Serendibsuthepica* Deeleman-Reinhold, 2001; **Location:** country: China; stateProvince: Yunnan Province; county: Jinghong; locality: Wild Elephant Valley; verbatimElevation: 814m; verbatimLatitude: 22°10′25.05″N; verbatimLongitude: 100°51′19.07″E; **Event:** year: 2022; month: 6; day: 5; **Record Level:** institutionID: the Museum of Hebei University; institutionCode: MHBU

#### Description

**Figure 5.**
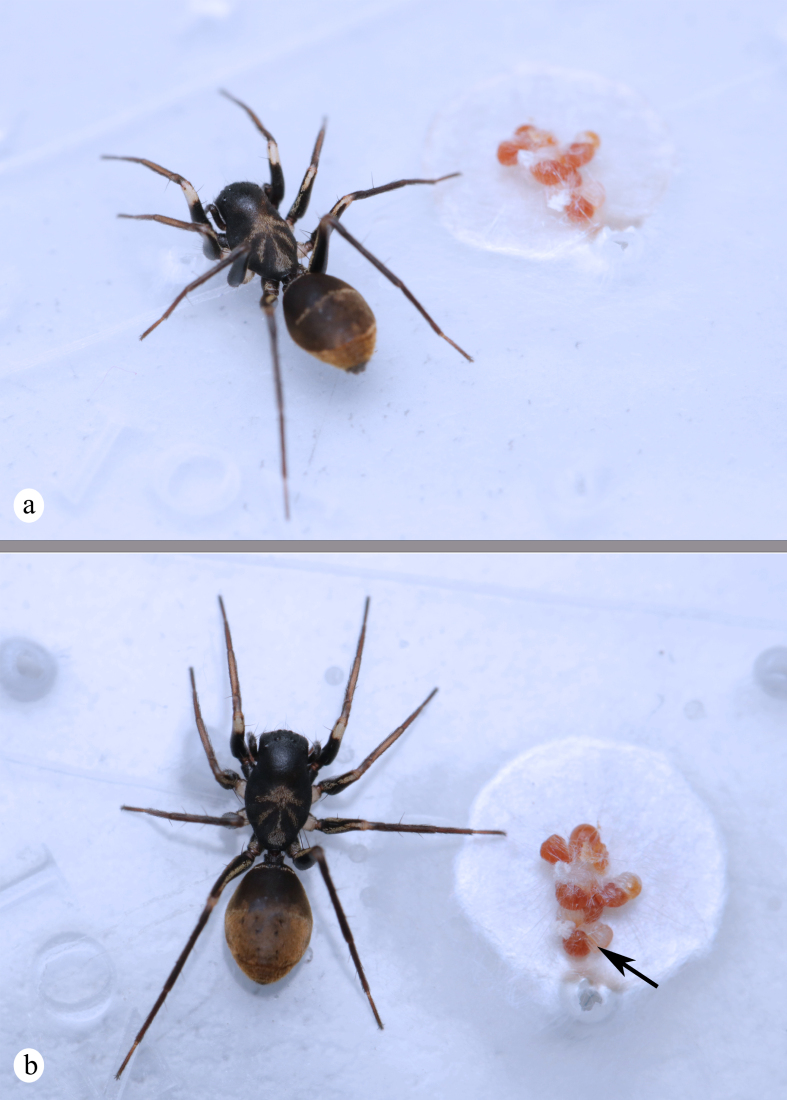
Living habitus of *Serendibsuthepica* Deeleman-Reinhold, 2001: **a**–**b** female, incomplete egg capsule and eggs (black arrows) (photographs by Kun Yu).

**Figure 6.**
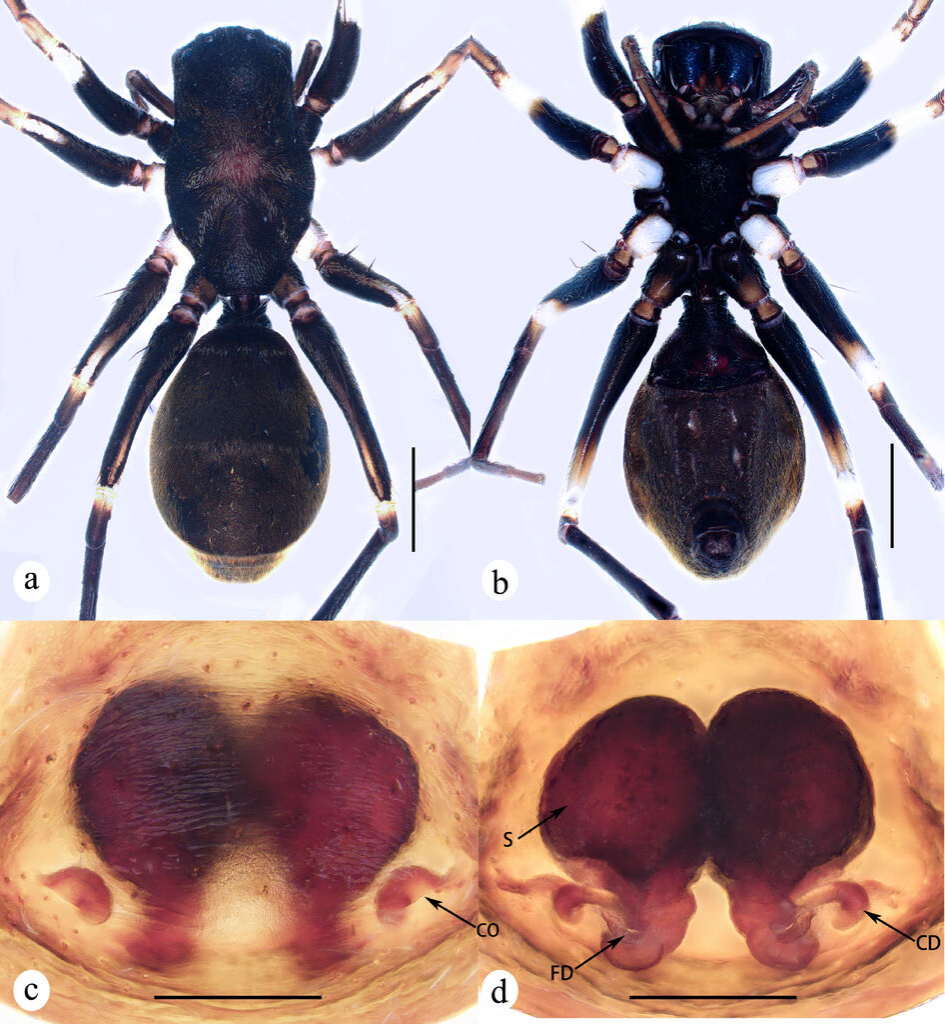
*Serendibsuthepica* Deeleman-Reinhold, 2001: **a** female habitus, dorsal view; **b** same, ventral view; **c** epigyne, ventral view; **d** vulva, dorsal view. Abbreviations: CO—copulatory opening; CD—copulatory duct; FD—fertilization duct; S—spermatheca. Scale bars: 1 mm (a–b); 0.2 mm (c–d).

Female (Fig. 5; Fig. 6a–b). Total length 5.43; carapace 2.78 long, 1.52 wide; abdomen 2.65 long, 1.82 wide. Eye sizes and interdistances: AME 0.10, ALE 0.09, PME 0.09, PLE 0.08; AME–AME 0.30, AME–ALE 0.15, ALE–ALE 0.60, PME–PME 0.42, PME–PLE 0.37, PLE–PLE 1.04, ALE–PLE 0.38. MOA 0.26 long, anterior width 0.42, posterior width 0.51. Clypeal height 0.23. Labium 0.17 long, 0.33 wide. Sternum 1.10 long, 0.86 wide. Measurements of legs: I 4.97 (1.51, 0.47, 1.16, 1.04, 0.79), II 4.82 (1.51, 0.42, 1.20, 1.02, 0.67), III 3.78 (1.50, 0.45, 1.19, 1.05, 0.59), IV 6.82 (2.20, 0.57, 1.70, 1.52, 0.83).

Carapace black, elongated, covered plumose hair (Fig. 6a). Legs slender, with spines; coxae II–III white, others black; Legs I–II brown, femora black, distally with white stripes; Legs III–IV black, femur III with one white stripe distally, femur IV with one thin yellowish stripe distally (Fig. 6a–b); femora I–II with five bristles ventrally, femur III with two, femur IV with one (Fig. 3b). Abdomen black, oval, covered golden hairs; with transverse strips anteriorly and medially; with short and grooved collar (Fig. 6a; Fig. 1b). Dorsal scutum large, anterior with two pairs of strong spines (Fig. 6a).

Epigyne as in Fig. 6c–d. Copulatory openings distinct, with straight sclerotized margins. Copulatory ducts transverse, coiled, gradually extended into large, black anterior spermathecae. Posterior spermathecae diverging, with ovate distal parts and connecting with fertilization ducts.

#### Diagnosis

See the diagnosis of *S.hispida* **sp. n.**.

#### Distribution

Thailand, Indonesia (Bali), China (Yunnan) (Fig. 9).

### Serendib muadai

Jäger, Nophaseud & Praxaysombath, 2012

8E66ADAD-AC20-5D29-AE2C-7013831B56B3

#### Description

##### Diagnosis and descriptions

Diagnosis, descriptions and illustration are provided by Jäger et al. (2012).

#### Distribution

Laos (Fig. 9).

### Serendib hispida

Zhang & Zhang sp. n.

7FA6DCEA-5704-5808-B167-78E4E745BEAA

7801B957-F982-4D6A-9874-88A34CA86AAC

#### Materials

- **Type status:** Holotype. **Occurrence:** recordedBy: Zhizhong Gao; individualCount: 1; sex: male; lifeStage: adult; occurrenceID: D008FE69-6F16-505D-B06B-614E3D6B23E8; **Taxon:** scientificName: *Serendibhispida*; **Location:** country: Malaysia; county: Negeri Pahang; locality: Karak; verbatimElevation: 68m; verbatimLatitude: 3°25′53.88″N; verbatimLongitude: 102°3′40.74″E; **Event:** year: 2015; month: 10; day: 27; **Record Level:** institutionID: the Museum of Hebei University; institutionCode: MHBU
- **Type status:** Paratype. **Occurrence:** recordedBy: Zhizhong Gao; individualCount: 1; sex: female; lifeStage: adult; occurrenceID: FAB32914-FE13-5027-8608-2F3886291DD7; **Taxon:** scientificName: *Serendibhispida*; **Location:** country: Malaysia; county: Negeri Pahang; locality: Pahang; verbatimElevation: 68m; verbatimLatitude: 3°25′53.88″N; verbatimLongitude: 102°3′40.74″E; **Event:** year: 2015; month: 10; day: 27; **Record Level:** institutionID: the Museum of Hebei University; institutionCode: NHBU

#### Description

**Figure 7.**
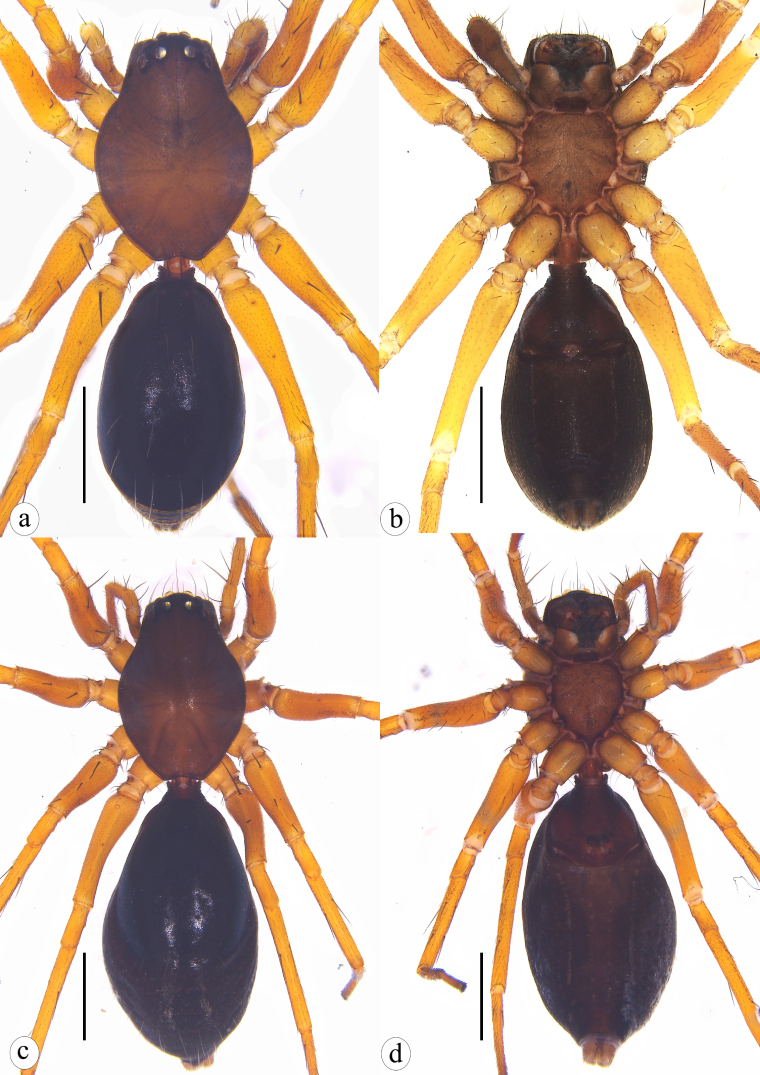
*Serendibhispida* **sp. n.**: **a** male habitus, dorsal view; **b** same, ventral view; **c** female habitus, dorsal view; **d** same, ventral view. Scale bars: 1 mm (a–d).

Male (Holotype) (Fig. 7a–b). Total length 4.02; carapace 1.87 long, 1.29 wide; abdomen 2.15 long, 1.23 wide. Eye sizes and interdistances: AME 0.11, ALE 0.09, PME 0.10, PLE 0.08; AME–AME 0.19, AME–ALE 0.13, ALE–ALE 0.42, PME–PME 0.25, PME–PLE 0.17, PLE–PLE 0.56, ALE–PLE 0.20. MOA 0.18 long, anterior width 0.28, posterior width 0.35. Clypeal height 0.12. Labium 0.10 long, 0.27 wide. Sternum 0.86 long, 0.77 wide. Measurements of legs: I 4.68 (1.34, 0.47, 1.08, 0.96, 0.83), II 4.61 (1.35, 0.34, 1.12, 0.99, 0.81), III 4.43 (1.29, 0.42, 1.03, 1.04, 0.65), IV 5.87 (1.65, 0.47, 1.36, 1.45, 0.94).

Carapace brown, wedge-shaped, with smooth surface. Legs slender, orange (Fig. 7a–b); femur I with six bristles ventrally, femur II with five, femur III with two, femur IV with one (Fig. 3a). Abdomen dark brown, oval, with dorsal scutum; with short, V-shaped and grooved collar. Dorsal scutum large, oval; anterior with two pairs of strong spines and posterior with two rows of white, erected setae (Fig. 7a, c; Fig. 2a, c). Epigastric sclerite extending anteriorly and sclerotized. Ventral scutum rectangular and heavily sclerotized (Fig. 7b).

**Figure 8.**
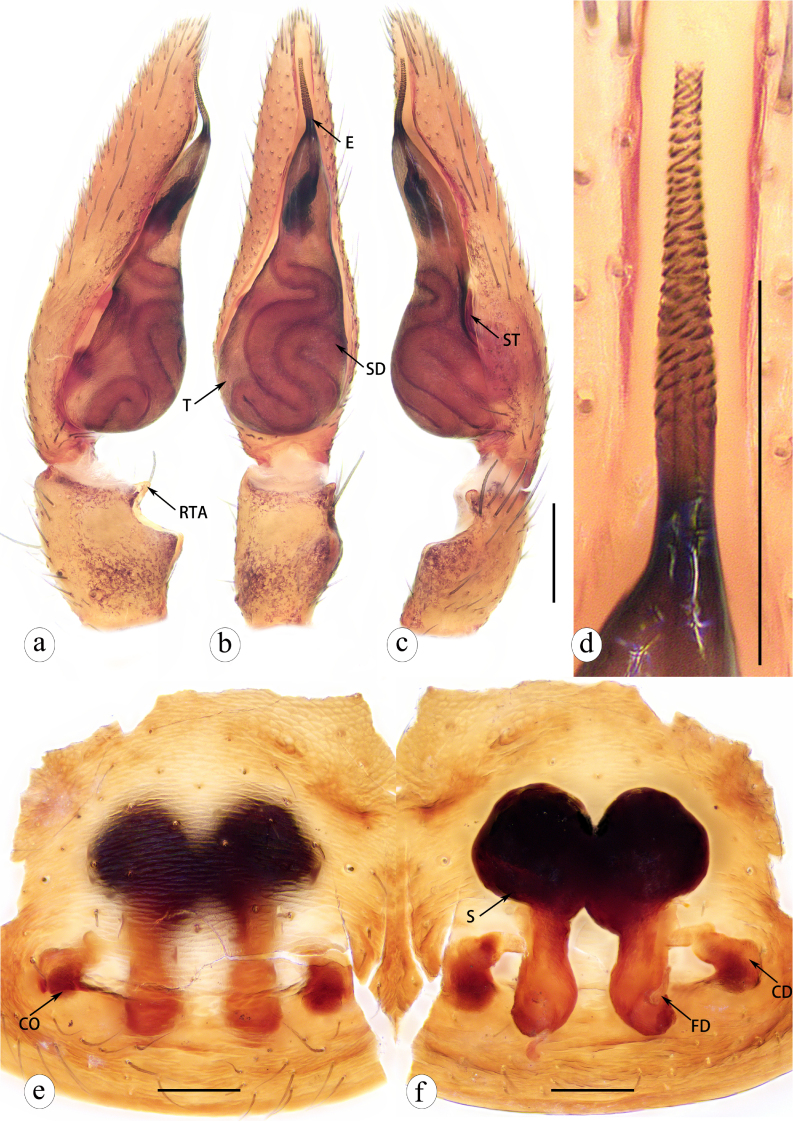
*Serendibhispida* **sp. n.**: a male left palp, prolateral view; **b** same, ventral view; **c** same, retrolateral view; **d** enlarged emblous, ventro-retrolateral view; **e** epigyne, ventral view; **f** vulva, dorsal view. Abbreviations: E—embolus; T—tegulum; ST—subtegulum; SD—sperm duct; RTA—retrolateral tibial apophysis; CO—copulatory opening; CD—copulatory duct; FD—fertilization duct; S—spermatheca. Scale bars: 0.1 mm (a–f).

Palp as in Fig. 8a–d. RTA subuliform, small. Sperm duct coiled and formed several loops; anterior with transverse U-shaped loop, middle double S-shaped, posterior with extra loop. Embolus straight spine-like process, with cataphracted stripes on surface.

Female (Fig. 7c–d). Total length 5.32; carapace 2.07 long, 1.44 wide; abdomen 3.25 long, 1.76 wide. Eye sizes and interdistances: AME 0.13, ALE 0.10, PME 0.11, PLE 0.09; AME–AME 0.22, AME–ALE 0.14, ALE–ALE 0.47, PME–PME 0.29, PME–PLE 0.21, PLE–PLE 0.65, ALE–PLE 0.18. MOA 0.22 long, anterior width 0.31, posterior width 0.37. Clypeal height 0.13. Labium 0.12 long, 0.31 wide. Sternum 0.93 long, 0.81 wide. Measurements of legs: I 5.18 (1.46, 0.46, 1.22, 1.11, 0.93), II 5.01 (1.44, 0.47, 1.17, 1.05, 0.88), III 4.84 (1.43, 0.45, 1.12, 1.11, 0.73), IV 6.32 (1.83, 0.49, 1.53, 1.55, 0.92). Other characteristics as in the holotype, except dorsal scutum extending about half the length of abdomen.

Epigyne as in Fig. 8e–f. Copulatory openings posteriorly situated and separated. Copulatory ducts long, stair-stepping and extended into black anterior spermathecae. Posterior spermathecae slender, subcylindrical, separated. Fertilization ducts short, semi-circular, lying the posterior spermathecae.

#### Diagnosis

The new species resembles that of *S.suthepica* in anterior abdomen with two pairs of strong spines, but can be distinguished by the following characteristics: 1) the longer embolus (vs. short in *S.suthepica*) (cf. Fig. 8a–d with figs 517–519 in Deeleman-Reinhold (2001)); 2) carapace brown and smooth (vs. black, elongated, covered yellowish plumose in *S.suthepica*) (cf. Fig. 7a, c with Fig. 6a); 3) legs orange (vs. brown or black with white stripes distally in *S.suthepica*) (cf. Fig. 7 with Fig. 6a–b); 4) copulatory ducts long, transverse and stair-stepping (vs. transverse, coiled in *S.suthepica*) (cf. Fig. 8e–f with Fig. 6c–d).

#### Etymology

The specific name is an adjective and refers to the characters of the dorsal scutum with two rows of setae. Latin *hispida* = hispid.

#### Distribution

**Figure 9.**
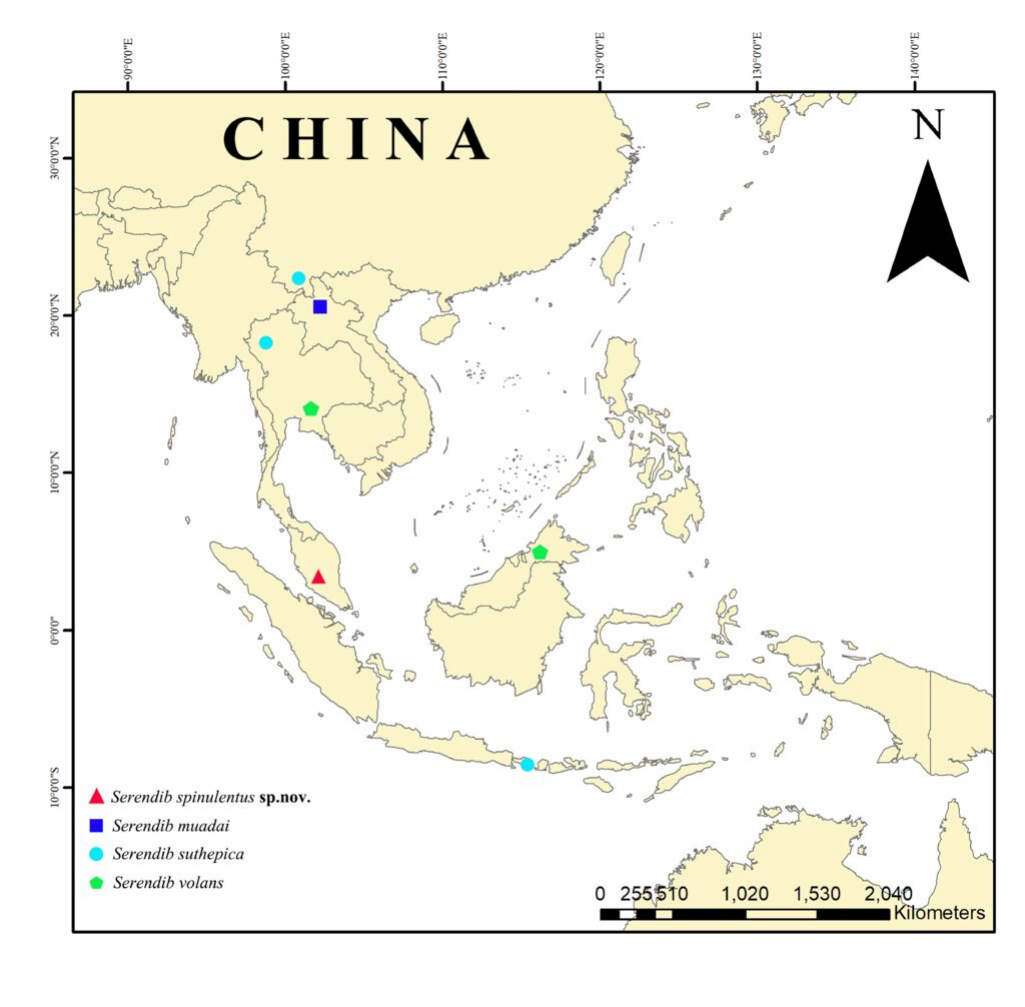
Distribution map of the genus *Serendib*.

Malaysia (Pahang) (Fig. 9).

## Supplementary Material

XML Treatment for Serendib

XML Treatment for Serendib volans

XML Treatment for Serendib suthepica

XML Treatment for Serendib muadai

XML Treatment for Serendib hispida

## Figures and Tables

**Figure 1a.**
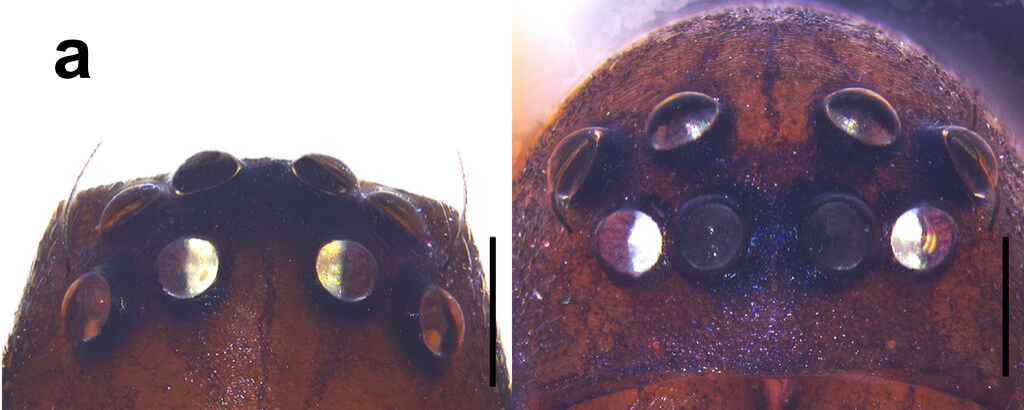
*Serendibhispida* **sp. n.**: male ocular area, dorsal view (left) and frontal view (right);

**Figure 1b.**
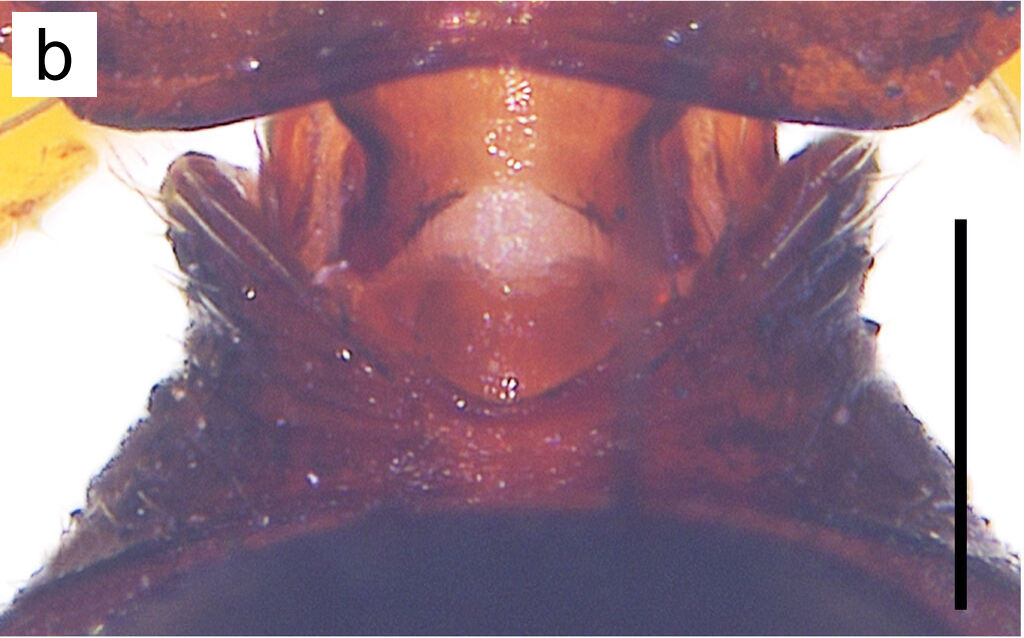
Same, female collar;

**Figure 1c.**
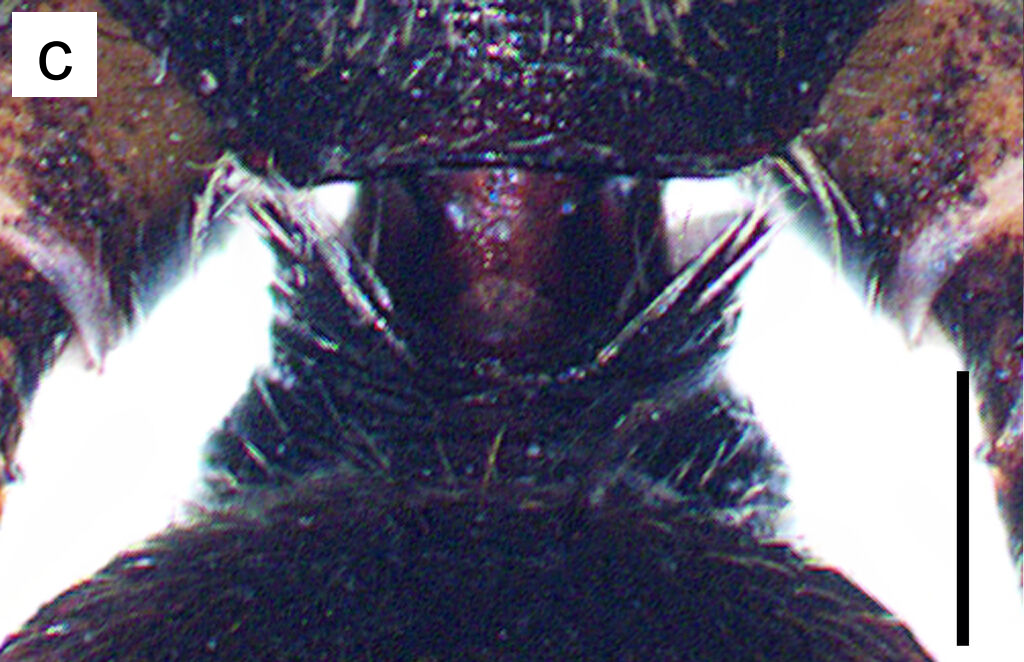
*Serendibsuthepica* Deeleman-Reinhold, 2001: female collar;

**Figure 1d.**
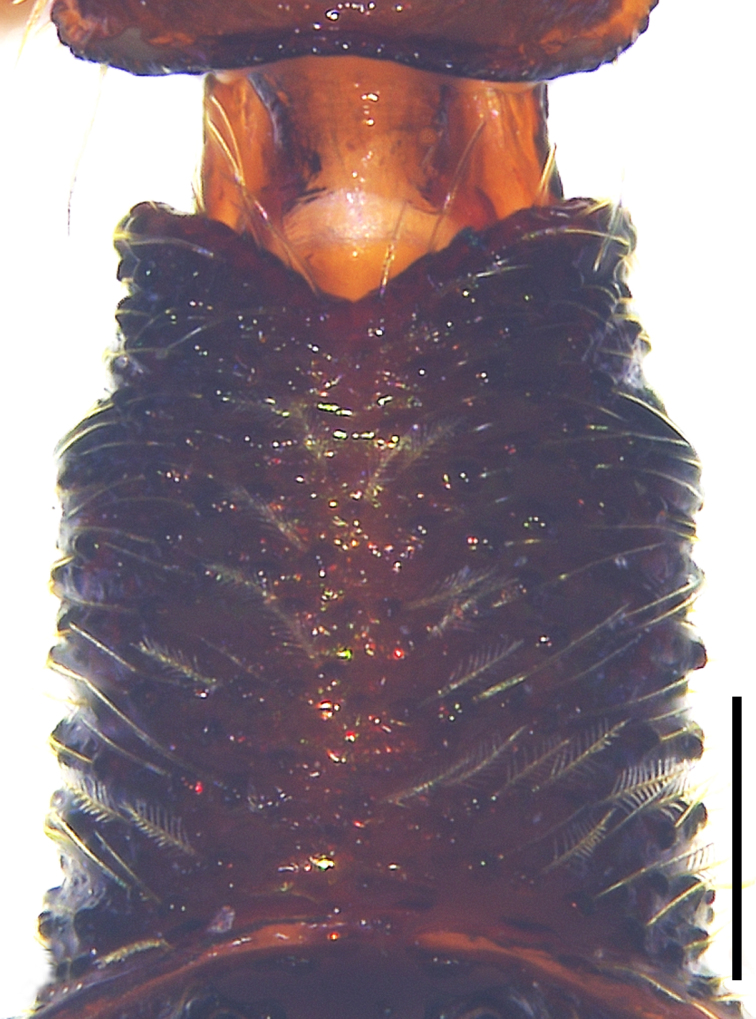
*Serendibvolans* Deeleman-Reinhold, 2001: female collar.

**Figure 2a.**
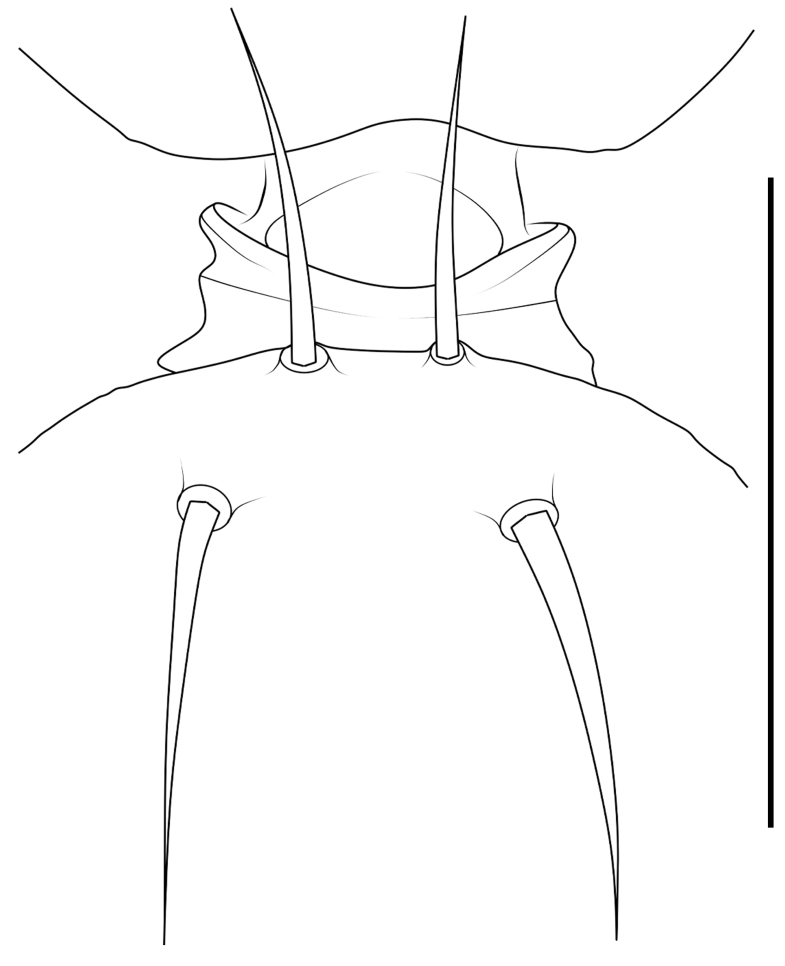
*Serendibhispida* **sp. n.**, dorsal view;

**Figure 2b.**
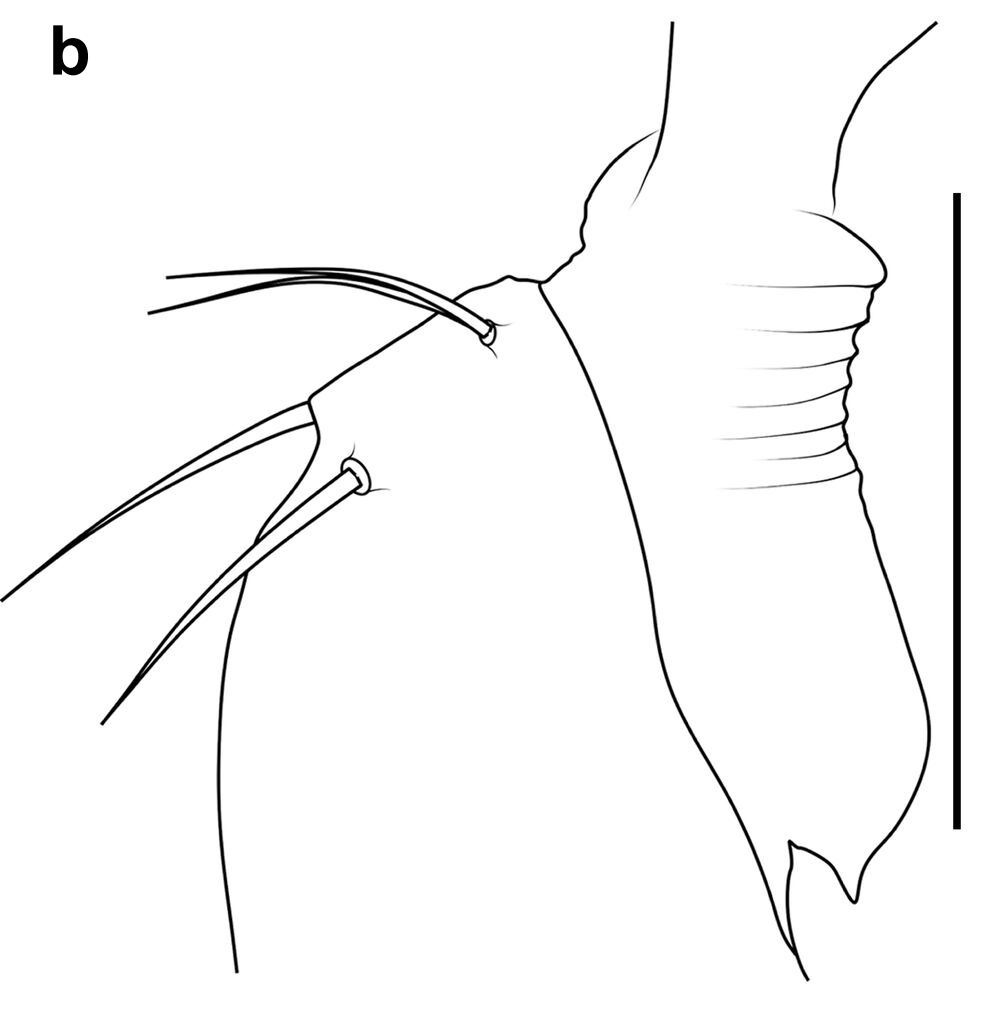
same, lateral view;

**Figure 2c.**
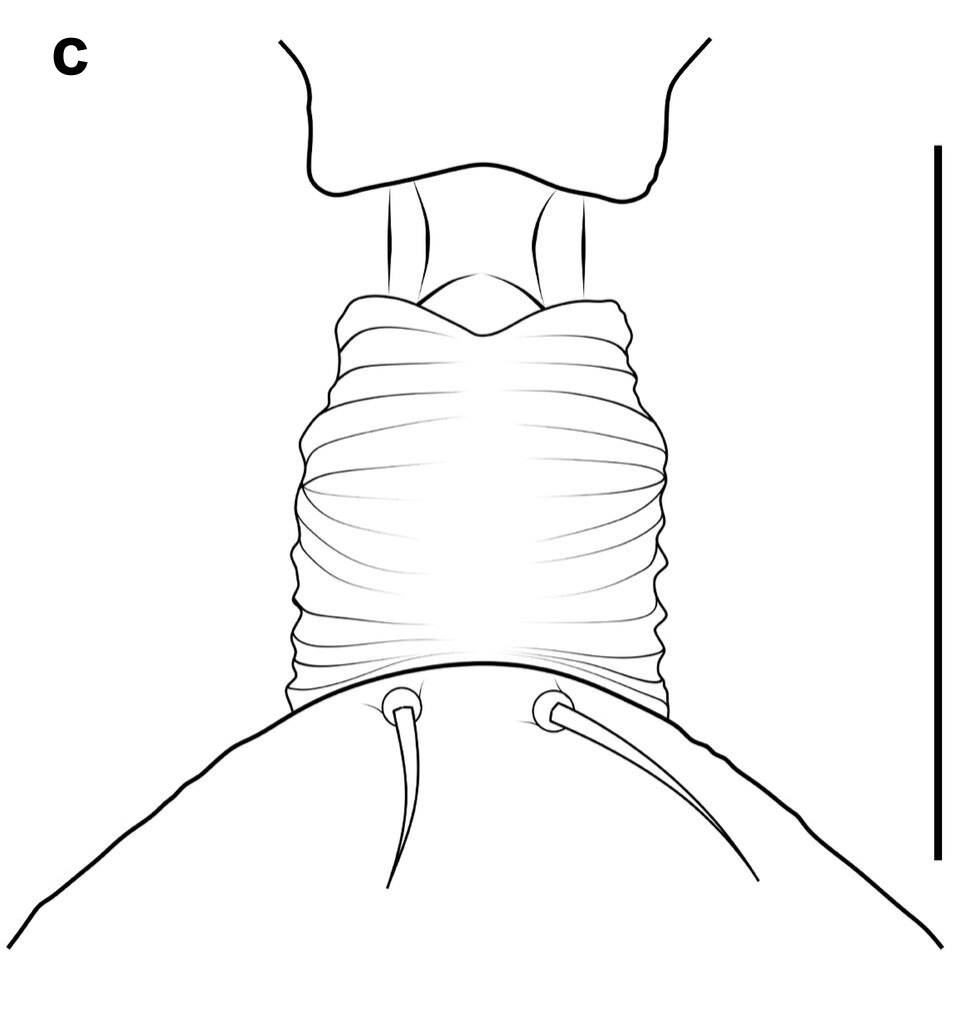
*Serendibvolans* Deeleman-Reinhold, 2001, dorsal view;

**Figure 2d.**
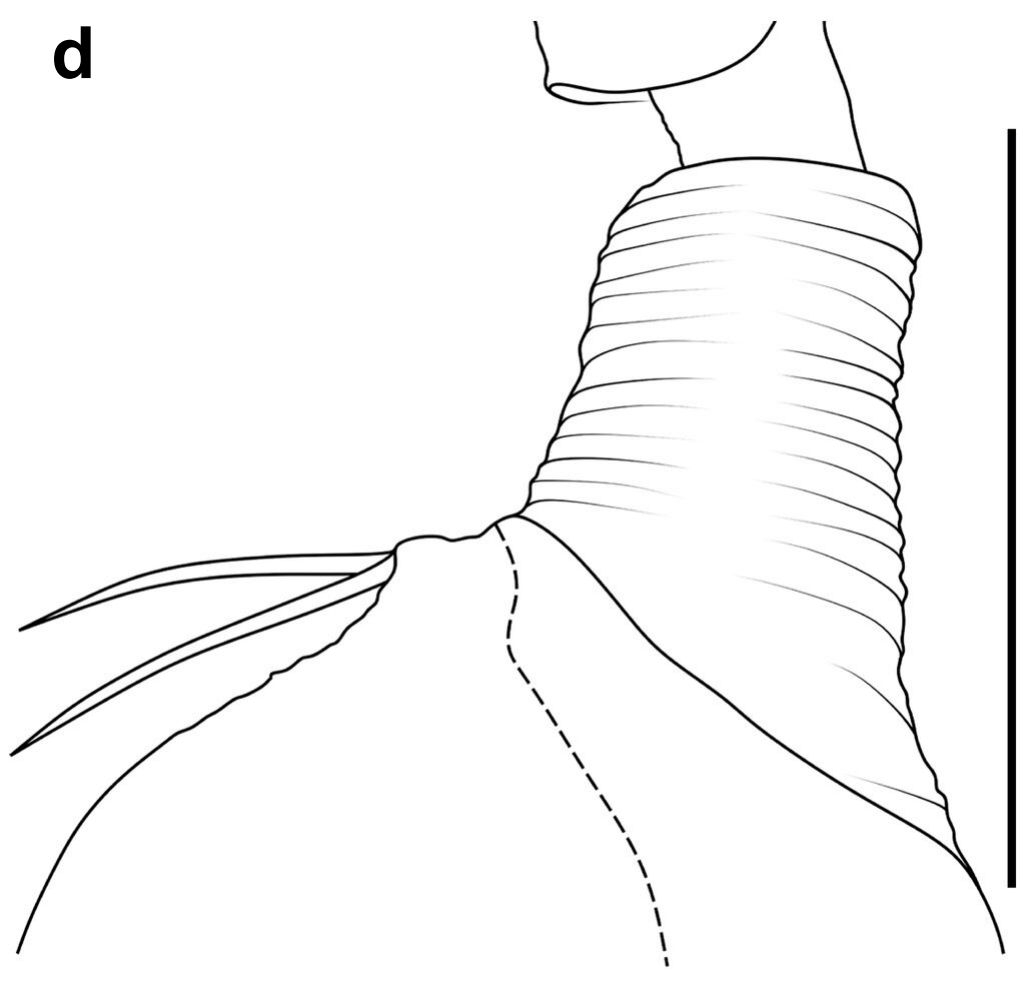
same, lateral view.

**Figure 3a.**
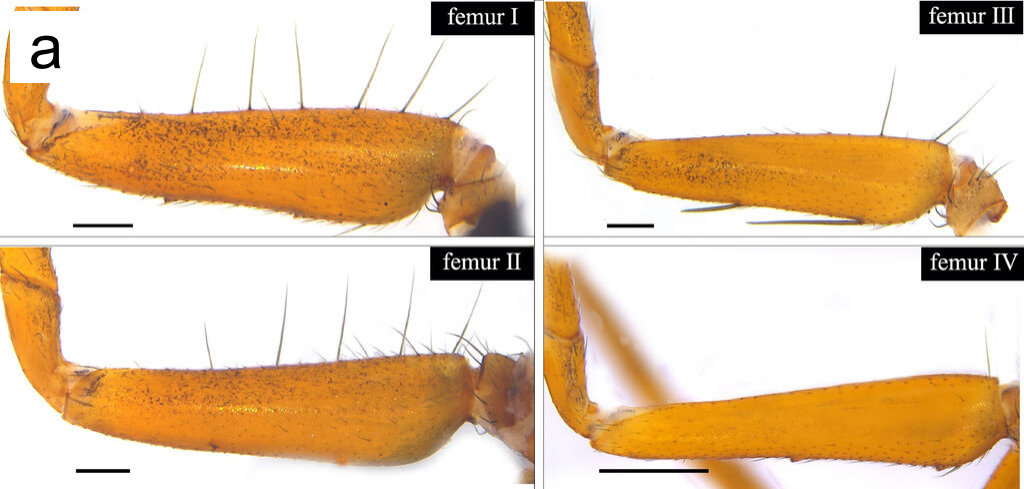
*Serendibhispida* **sp. n.**;

**Figure 3b.**
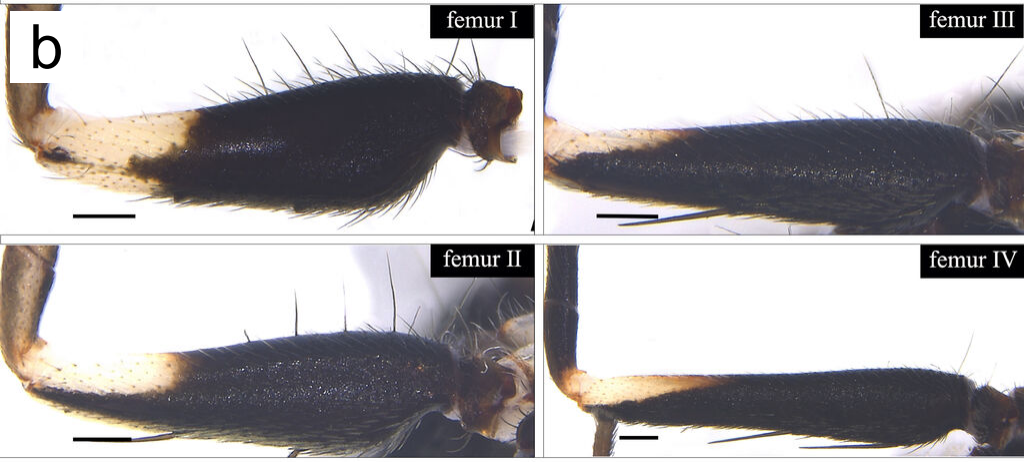
*Serendibsuthepica* Deeleman-Reinhold, 2001;

**Figure 3c.**
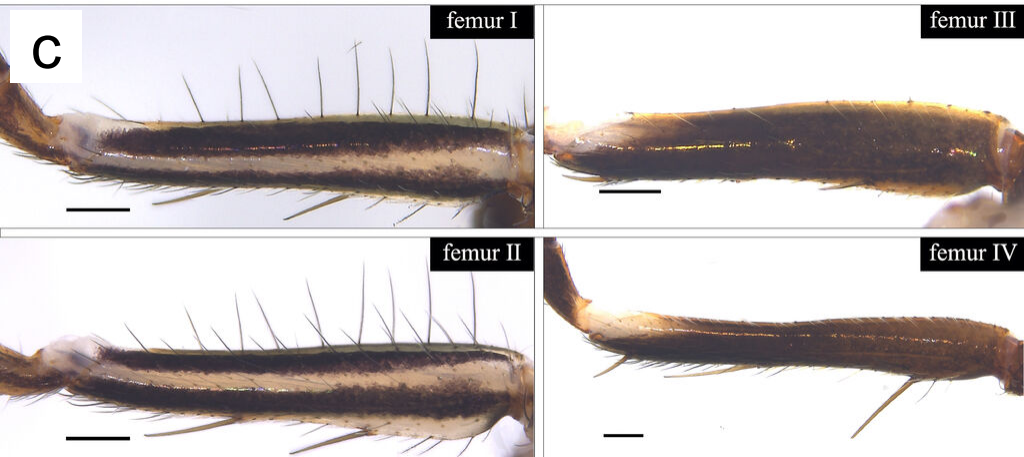
*Serendibvolans* Deeleman-Reinhold, 2001.
